# Low Seroprevalence of *Toxocara* Infection in Schizophrenic Inpatients in Durango, Mexico: A Case Control Study

**Published:** 2014-12

**Authors:** Cosme Alvarado-Esquivel, Jesús Hernández-Tinoco, Luis Francisco Sánchez-Anguiano, Jorge Arturo Cisneros-Martínez

**Affiliations:** 1 Biomedical Research Laboratory, Faculty of Medicine and Nutrition, Juárez University of Durango State, Durango, Mexico;; 2 Institute for Scientific Research, Juárez University of Durango State, Avenida Universidad S/N. 34000 Durango, Dgo. Mexico

**Keywords:** Case-control study, epidemiology, schizophrenia, seroprevalence, toxocariasis

## Abstract

Psychiatric patients have a higher seroprevalence of toxocariasis than general population. However, there is poor knowledge about any specific psychiatric diagnosis associated with toxocariasis. The aim of the study was to determine whether seropositivity to *Toxocara* was associated with schizophrenia. Through an age and gender-matched case-control seroprevalence study in Durango City, Mexico, 50 schizophrenic inpatients in a public psychiatric hospital and 100 control subjects of the general population were compared for the presence of anti-*Toxocara* IgG antibodies. One of the 50 (2%) schizophrenic inpatients, and 3 (3%) of the 100 controls were positive for anti-*Toxocara* IgG antibodies. No statistically significant difference in *Toxocara* seroprevalence among cases and controls was found (*P*=0.59). The *Toxocara* positive schizophrenic patient suffered from paranoid schizophrenia (F20.0) and had a number of putative risk factors for *Toxocara* exposure including contact with cats, dogs and other animals, worked in agriculture, and consumed undercooked meat, unwashed fruits and vegetables, and untreated water. Results suggest that seroprevalence of *Toxocara* infection was low and not associated with schizophrenia in psychiatric inpatients in Durango, Mexico. However, further studies to elucidate the association of toxocariasis with schizophrenia are needed.

## INTRODUCTION

*Toxocara *is a parasite causing infections in humans around the world (1, 2). Infections may occur by ingesting *Toxocara* eggs shed by infected dogs and cats in the environment (3, 4). In addition, infection with *Toxocara* may occur by ingesting *Toxocara* larvae from undercooked giblets (5). *Toxocara* disseminates via the bloodstream to anywhere in the host body including liver, lungs, muscles, eyes, and central nervous system (2, 6). Although most infections with *Toxocara *are asymptomatic, a serious illness and death may occur in some infected individuals (1, 2, 7). Ocular toxocariasis may lead to permanent vision loss (8). The epidemiology of *Toxocara* infection in psychiatric patients in general and in schizophrenic patients in particular is poorly understood. Some studies have found an increased seroprevalence of toxocariasis in psychiatric patients (9, 10). However, it is not clear whether any specific psychiatric diagnosis is associated with toxocariasis. The present study adds new information to a previous work (10) where seroprevalence to *Toxocara* was found higher in psychiatric patients than in control subjects. However, differences among the present study and the previous study (10) exist including the aims and study populations. The aim of the previous study (10) was to determine the seroprevalence of *Toxocara* infection in psychiatric inpatients while in the present study the aim was to determine the association of toxocariasis with schizophrenic inpatients. In the present study, a new population of schizophrenic patients was included.

## METHODS

### Selection and description of participants

This case-control study was performed using serum samples from recent *Toxoplasma gondii* serosurveys in Durango City, Mexico (11, 12). Fifty schizophrenic inpatients attended in the Mental Health Hospital in Durango City (11) and 100 control subjects without history of schizophrenia of the general population in the same city (12) were studied. Of the 50 schizophrenic patients, 40 were male and 10 female. Patients were 18-72 years old (mean 45.12 ± 11.5 years) and suffered from a number of schizophrenic disorders including paranoid schizophrenia (F20.0) (n=33), catatonic schizophrenia (F20.2) (n=1), undifferentiated schizophrenia (F20.3) (n=11), post-schizophrenic depression (F20.4) (n=1), residual schizophrenia (F20.5) (n=2), and simple schizophrenia (F20.6) (n=2). Patients had suffered from schizophrenia from few months (2 patients) until 43 years. Control (n=100) subjects were matched with schizophrenic patients by age and gender. The mean age in controls was 45.5 ± 13.1 (range: 18-72) and comparable with that in schizophrenic patients (*P*=0.70).

Participation in this study was voluntary. This study was approved by the Institutional Ethical Committee of the Hospital of Mental Health “Dr. Miguel Vallebueno” of the Secretary of Health in Durango City. Only archival serum samples from previous surveys (11, 12) were used. In such previous studies, the purpose and procedures of the surveys were explained to all participants, and a written informed consent was obtained from each subject. We used a standardized questionnaire to explore socio-demographic, clinical and behavioral characteristics of the participants.

### Technical information

Serum samples of the participants were analyzed for anti-*Toxocara* IgG antibodies with a commercially available enzyme immunoassay (EIA) “*Toxocara”* kit (Diagnostic Automation, Inc. Calabasas, CA, U.S.A.). Absorbance reading equal to or greater than 0.3 OD units was considered positive. All EIA tests were performed following the instructions of the manufacturer.

### Statistics

The statistical analysis was performed with the aid of the software Epi Info version 3.5.4. Age among cases and controls was compared by the student’s *t* test. The Fisher exact test was used for comparison of the frequencies among groups. The criterion for statistical significance was set at *P* less than 0.05.

## RESULTS

One of the 50 (2%) schizophrenic inpatients, and 3 (3%) of the 100 controls were positive for anti-*Toxocara* IgG antibodies (*P*=0.59). Stratification by gender showed that one (2.5%) of the 40 male patients and 3 (3.8%) of the 80 male controls were positive for anti-*Toxocara* antibodies (*P*=0.59). None of the female patients and female controls was positive for anti-*Toxocara* antibodies. The *Toxocara* positive schizophrenic patient was a male aged 37 years old suffering from paranoid schizophrenia (F20.0). He had had cats, dogs and other animals and worked in agriculture. He had consumed undercooked meat, unwashed fruits and vegetables, and untreated water.

## DISCUSSION

In the present study, a similar seroprevalence of *Toxocara* seropositivity in schizophrenic inpatients than in control subjects was found. Therefore, this finding does not support an association between toxocariasis and schizophrenia. Our results conflict with those reported in a Turkish study where researchers found that *Toxocara* seroprevalence was higher in patients suffering from schizophrenia (45.9%) than in control subjects (2%) (13). The conflicting results about the association of *Toxocara* seropositivity and schizophrenia among the studies might be explained by differences in the characteristics of the study populations. Patients in the present study were urban while most of the patients studied in the Turkish study were rural. The only one schizophrenic patient with anti-*Toxocara* antibodies in the present study had certainly risk factors for infection including contact with cats and dogs, consumption of undercooked meat, unwashed raw vegetables and fruits and soil contact. The low seroprevalence of *Toxocara* exposure in schizophrenic patients found contrasts with those reported in other populations in the region including 13% in waste pickers (14), and 26.2% in persons of Tepehuanos ethnicity (15). Differences in seroprevalence among the studies might be explained by differences in the characteristics of the studied populations. Tepehuanos are mostly rural and the schizophrenic patients studied were urban. The sanitation and hygiene practices might be lower in waste pickers than in schizophrenic inpatients. The very low number of positive schizophrenic patients with *Toxocara* exposure found in the present study did not allow us to perform further statistical analyses to identify socio-demographic, clinical and behavioral characteristics associated with *Toxocara* exposure. The low seroprevalence of *Toxocara* exposure found in schizophrenic patients suggests that such exposure might have a minor epidemiological importance in schizophrenia in our region. Other infections may have a more important role in schizophrenia than *Toxocara* exposure in our region, for instance, infections with *Toxoplasma gondii* (11).

We concluded that seroprevalence of *Toxocara* exposure was low and not associated with schizophrenia in psychiatric inpatients in Durango City, Mexico. Further studies to elucidate the association of toxocariasis with schizophrenia are needed.
